# Global Spread of Coronavirus Disease 2019 and Malaria: An Epidemiological Paradox in the Early Stage of A Pandemic

**DOI:** 10.3390/jcm9041138

**Published:** 2020-04-16

**Authors:** Pietro Emanuele Napoli, Matteo Nioi

**Affiliations:** 1 Department of Surgical Science, Eye Clinic, University of Cagliari, 09124 Cagliari, Italy; 2 Department of Clinical Sciences and Public Health, Forensic Medicine Unit, University of Cagliari, 09124 Cagliari, Italy

**Keywords:** COVID-19, coronavirus disease, malaria, antimalarials, ACE-2 receptor, epidemiological paradox

## Abstract

In the current work, we discovered and analyzed the epidemiological paradox between coronavirus disease 2019 (COVID-19) and malaria in the initial phase of the ongoing pandemic. From the analysis of distribution data, the endemic presence of malaria seems to protect some populations from COVID-19 outbreak, particularly in the least developed countries. In this sense, molecular and genetic variations associated with malaria (e.g., in ACE2) might play a protective role against coronavirus infection. Moreover, the mechanism of action of some antimalarial drugs, e.g., the antiviral function, suggests their potential role in the chemoprophylaxis of coronavirus epidemics, despite possible adverse effects (e.g., retinal toxicity). All these data provide important insights to understand the spreading mechanisms of COVID-19, and to direct scientific research toward the study of some currently available medications.

## 1. Introduction

The recent spread of coronavirus disease 2019 (COVID-19) constitutes an important and unsolved public health problem with potentially serious economic and social consequences [1].

Scholars and the World Health Organization (WHO) have expressed great concerns about the global pandemic and, in particular, regarding the involvement of the poorest African countries. In the latter, the scarce economic resources, the weakness of the health system, and the endemic presence of diseases associated with immunodeficiency (i.e., AIDS) may make the containment of the outbreak particularly difficult [2]. 

## 2. Brief Summary of the Evidence and Related Implications

The aforementioned concern is also justified by the fact that, in the recent past, some types of betacoronaviruses (Beta-CoVs), such as the Middle East respiratory syndrome-related coronavirus (MERS-CoV), have proven to be resistant to elevated temperatures and reduced relative humidity, thus causing a considerable number of deaths during the summer season in tropical areas where temperatures reached 40 °C [3]. Moreover, the spread of COVID-19 also occurred in areas with tropical temperatures, e.g., in the province of Guangdong (23°24′N 113°30′E), or in countries near to the Equator such as Malaysia (Kuala Lampur, 3°8′N 101°41′E).

The novel Beta-CoV responsible for COVID-19, named “severe acute respiratory syndrome coronavirus 2” (SARS-CoV-2), may have some characteristics in common with MERS-CoV and SARS-CoV, representing a different species (viral lineage B) within the same *genus* [4]. Interestingly, only 40% of amino acids of SARS-CoV-2 coincide with those of Beta-CoVs linked to SARS [5].

**Figure 1:**
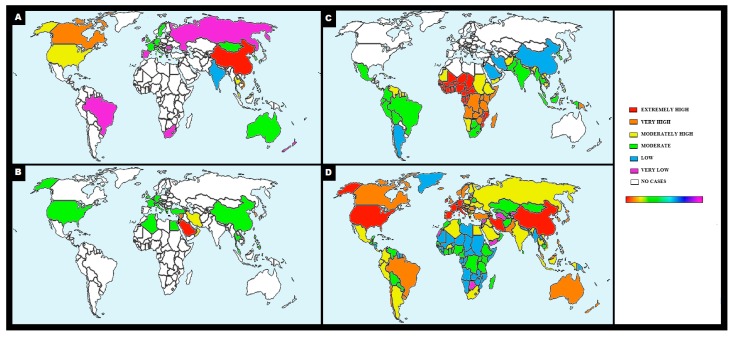
**Distribution maps of betacoronaviruses and malaria.** Color scale of the number of cases (white = none, light purple = very low, blue = low, green = moderate, yellow = moderately high, orange = very high, red = extremely high). Color bar indicates the sequence of positive values [very low number of cases = light purple to extremely high number of cases = red]. **Panel A** shows the map representing the spread of severe acute respiratory syndrome coronavirus 2 (**SARS-CoV)** in the terminal phase of epidemic in 2003 (WHO data source) [7]. The map in the **panel B** represents the confirmed global cases of Middle East respiratory syndrome-related coronavirus **(MERS-CoV)** 2012-2017 as of 1 September 2017 (WHO data source) [8]. **Panel C** represents the map of countries with indigenous cases of **malaria** in 2000 and their status by 2017 (WHO data source) [9].In **panel D** is shown the distribution of coronavirus disease 2019 (**COVID-19)** cases as of 25 March 2020 (WHO data source) [6].

Surprisingly, the historical data relating to SARS-CoVs and MERS-CoV epidemics reveal a limited or no dissemination of these disorders in African countries, despite the limitations of the health system and the geographical continuity of the two outbreaks (Figure 1A–D) [6]. Similarly, very recent data concerning the spread of COVID-19 in Africa describe a much smaller than expected number of cases, particularly when considering the vulnerability of this population (Figure 1D). Counterintuitively, even in India (which is a developing country relatively close to Wuhan) and in some countries of South America, a relatively limited number of cases of COVID-19 have occurred during the approximately three months of the ongoing pandemic [6].

Although we cannot exclude an underestimation of the real cases of SARS-CoV-2 epidemic in poorest countries of Africa due to the lack of appropriate diagnostic techniques, no official report has documented an increase in the death rate for pneumonia of unknown causes. Accordingly, alternative hypotheses can be derived from epidemiological data to explain the disproportional spread of the disease. 

An absolutely curious element regards the comparison between the distribution maps of Beta-CoVs diseases (i.e., MERS, SARS, and COVID-19) and malaria through which it is possible to reveal an inverse relationship in the overall number of cases infected with coronaviruses and Plasmodium parasite (Figure 1A–D) [9]. 

As of 28 March 2020, 10:00 (CET), i.e., after approximately three months of pandemic, merely 247 total cases of COVID-19 (with six deaths) have been detected in the six African countries with the highest number of malaria cases, which are defined by the World Malaria Report 2019 (Figure 1D, WHO data source). Specifically, in the latter document are included the following States (~400 million of people) associated with a variable percentage of malaria patients out of global cases: Nigeria (25%), the Democratic Republic of the Congo (12%), Uganda (5%), Ivory Coast (4%), Mozambique (4%), and Niger (4%).By adding Ethiopia, South Africa, and India, the number of deaths and cases associated with COVID-19 increases to 25 and 2,156, respectively, out of a total of ~2 billion people (Figure 1D, WHO data source). 

As a comparison, in the WHO European region (~741 million of people), the number of COVID-19 cases and deaths has been 324,343 and 18,740, respectively. Meanwhile, 85,228 cases of COVID-19 and 1243 deaths have been reported in the United States of America (~327 million of people) (Figure 1D, WHO data source).

## 3. Hypotheses and Discussion

The assumption that malaria has a protective effect against recent Beta-CoVs epidemics seems to be based on two main premises. 

Firstly, the presence of an evolutionary adaptation related to malaria (in the endemic areas or in those where it has been eradicated) might play a role in limiting the spread of COVID-19. Accordingly, some variants of the ACE2 receptor, which is used by the coronavirus to infect cells, may protect these populations [10,11,12]. This phenomenon potentially explains the heterogeneous diffusion of COVID-19 among countries (e.g., Nigeria in comparison with South Africa) or within them (e.g., South Sardinia in comparison with Lombardy). Clearly, the available data in this sense are still scarce and additional genetic studies are necessary to confirm this hypothesis.

Secondly, a different discussion can be made regarding the treatment used for malaria, which widely involves the use of chloroquine and its derivatives (in particular, hydroxychloroquine). These drugs are currently used in Western countries, mainly for some autoimmune disorders, and monitored over the years, particularly to verify their ocular toxicity (‘chloroquine retinopathy’).

Of note, literature data on the efficacy of these drugs against Beta-CoVs syndromes have been scientifically evidenced by the first SARS epidemic [13,14].

More recently, some authors have reported the usefulness of hydroxychloroquine at a dosage of 400 mg per day for ten days in the treatment of symptomatic SARS-CoV-2 pneumonia [15]. The mechanism of action of these antimalarial drugs would result in an increase in endosomal pH by preventing the fusion between virus and cell, or interfering in the glycosylation process of cellular receptors of coronavirus. In addition, it is hypothesized that these antimalarials have an immunomodulating effect that contributes to increasing their efficacy in vivo [16]. 

Of note, chloroquine is commonly used in several countries in the treatment and prevention of malaria despite drug resistance or government recommendations (also through its distribution in unofficial channels, especially in sub-Saharan Africa), due to its low price and wide availability [17].

With similar characteristics to chloroquine, amodiaquine and mefloquine are also widely used in Africa as antimalarials with antiviral action against SARS-CoV and MERS-CoV [18]. Surprisingly, even some medications used in cases of chloroquine resistance (e.g., doxycycline, azithromycin) have demonstrated antiviral action against a large group of viruses [19].

It is of relevance to note that travelers planning a trip to some countries where malaria is endemic must take these drugs for prophylaxis, thus reducing the possibility of viral transmission. 

From all these considerations, it is possible to presume that the use of chloroquine, its derivatives, and other antimalarials may represent an unintentional chemoprophylaxis in African countries against Beta-CoVs epidemics, which potentially slows down the spread of COVID-19. 

Therefore, the involuntary achievement of an indirect protection from SARS-CoV-2 infection (similar to "herd immunity") for individuals who are not immune, in malaria countries, is likely to occur for different reasons. This slowdown of the epidemic in these countries may allow local populations to have extra time to implement all the rules for the prevention of contagion in an effective and timely manner.

Interestingly, although the low median age of Africans is expected to be associated with a reduced case fatality rate, the high presence of young adults in Africa does not eliminate the risk of contagion within the population or the possibility of detecting a certain number of cases.

Starting from the analysis of epidemiological data on malaria and on the effectiveness of antimalarials in the treatment of Beta-CoVs diseases, it is possible to state that scientific research is expected to verify the potential of antimalarials in reducing the spread of COVID-19 even in non-African countries. For instance, the effect of the administration of chloroquine derivatives in areas where malaria is endemic seems to preserve these geographic areas (i.e., the least developed countries) from COVID-19 outbreak, despite some rare side effects (e.g., retinal toxicity). 

In this sense, a drug repurposing-based approach for COVID-19 would have numerous advantages, including prompt availability (the time required for traditional drug development is discordant with the urgent need for novel treatments for coronavirus epidemic), more experience with regard to contraindications and side effects, and elimination of costs associated with de novo drugs discovery. A famous example of successful repurposed drug is Viagra (Pfizer) for erectile dysfunction (angina represents its original indication).

Testing these assumptions could have important implications for epidemic management strategies. Accordingly, at least 10 clinical trials are currently underway to verify the efficacy of chloroquine and its derivates for COVID-19 (e.g., NCT04261517, 2/14/20; ChiCTR2000029826, 2/2/20) [20]. Interestingly, hydroxychloroquine is a less toxic derivate of chloroquine that has demonstrated potential against Beta-CoVs [21]. 

In particular, it is important to understand whether the prophylactic administration of antimalarial medications in populations at risk may contribute to a significant containment of SARS-CoV-2 epidemic with important economic and social outcomes. Even the mere prophylactic administration of chloroquine or its derivatives to ‘exposed’ health workers would help to ensure greater serenity in emergency management. The advantages would undoubtedly include the immediate availability of a very low-cost drug, whose side effects (e.g., toxic retinopathy), potential therapeutic doses, and contraindications, in some subcategories of the population (i.e., G6PD deficiency, pregnancy, etc.), have long been known. 

## 4. Conclusions

In conclusion, all these data provide important insights to better understand the early spreading mechanisms of COVID-19, and to direct scientific research toward the study of the existing antimalarials drugs (e.g., chloroquine, amodiaquine, mefloquine, or doxycycline). Further studies should be carried out promptly to verify the hypothesis presented here without overlooking the innumerable advantages given by the implementation of a suitable strategy in the shortest possible time frame. Delaying such studies could lead to unbearable loss in terms of human lives and economic resources.

## Data and Materials Availability

A different abstract of the paper is present at the following address: https://papers.ssrn.com/sol3/papers.cfm?abstract_id=3554389 Nioi, Matteo and Napoli, Pietro Emanuele, Global Spread of Coronavirus Disease 2019 and Malaria: An Epidemiological Paradox. (March 10, 2020). Available at SSRN: https://ssrn.com/abstract=3554389 or http://dx.doi.org/10.2139/ssrn.3554389.
